# Reconstructing three-dimensional protein crystal intensities from sparse unoriented two-axis X-ray diffraction patterns

**DOI:** 10.1107/S1600576717006537

**Published:** 2017-06-14

**Authors:** Ti-Yen Lan, Jennifer L. Wierman, Mark W. Tate, Hugh T. Philipp, Veit Elser, Sol M. Gruner

**Affiliations:** a Laboratory of Atomic and Solid State Physics, Cornell University, Ithaca, NY 14853, USA; b Cornell High Energy Synchrotron Source (CHESS), Cornell University, Ithaca, NY 14853, USA; c Macromolecular Diffraction Facility at CHESS (MacCHESS), Cornell University, Ithaca, NY 14853, USA; d Kavli Institute for Nanoscale Science, Cornell University, Ithaca, NY 14853, USA

**Keywords:** X-ray serial microcrystallography, sparse data, EMC algorithm, protein microcrystallography, synchrotron radiation sources

## Abstract

Recently, there has been a growing interest in adapting serial microcrystallography (SMX) experiments to existing storage ring (SR) sources. For very small crystals, however, radiation damage occurs before sufficient numbers of photons are diffracted to determine the orientation of the crystal. The challenge is to merge data from a large number of such ‘sparse’ frames in order to measure the full reciprocal space intensity. To simulate sparse frames, a dataset was collected from a large lysozyme crystal illuminated by a dim X-ray source. The crystal was continuously rotated about two orthogonal axes to sample a subset of the rotation space. With the EMC algorithm [expand–maximize–compress; Loh & Elser (2009). *Phys. Rev. E*, **80**, 026705], it is shown that the diffracted intensity of the crystal can still be reconstructed even without knowledge of the orientation of the crystal in any sparse frame. Moreover, parallel computation implementations were designed to considerably improve the time and memory scaling of the algorithm. The results show that EMC-based SMX experiments should be feasible at SR sources.

## Abstract 2

To simulate the signal level of serial microcrystallography experiments at storage ring sources, data frames were collected from a large lysozyme crystal rotated about two orthogonal axes and illuminated by a dim X-ray source. Using the EMC algorithm, this study shows that three-dimensional intensity reconstruction is still feasible even without the knowledge of the crystal orientation in each data frame.

## 1. Introduction   

The advance of serial femtosecond microcrystallography (SFX) at X-ray free-electron lasers (XFELs) (Chapman *et al.*, 2011 ▸; Boutet *et al.*, 2012 ▸) allows structure determination with protein crystals whose sizes are too small for conventional crystallography experiments. SFX is based on injecting a sequence of randomly oriented microcrystals to intercept a train of X-ray pulses. The tens of femtoseconds long pulse width enables the photon scattering process to outrun the radiation damage of the crystals, while the ultra-high brightness of the pulses results in a sufficient number of resolvable Bragg peaks collected by a fast-framing detector (Philipp *et al.*, 2008 ▸) for indexing. Using this concept of ‘diffract before destroy’ (Neutze *et al.*, 2000 ▸), a complete dataset can be obtained given enough indexed data frames. SFX has the advantage of rapid data collection owing to the high repetition rate of the X-ray pulses and provides a promising means to study proteins that do not readily form large single crystals.

Despite the success of SFX, the paucity of XFEL beamtime has inspired interest in adapting serial microcrystallography (SMX) experiments to storage ring (SR) sources (Gati *et al.*, 2014 ▸; Stellato *et al.*, 2014 ▸; Heymann *et al.*, 2014 ▸; Nogly *et al.*, 2015 ▸; Botha *et al.*, 2015 ▸; Gruner & Lattman, 2015 ▸; Schubert *et al.*, 2016 ▸). Radiation damage cannot be outrun in the same way at SR sources, thereby much more strongly limiting the dose per protein that can be tolerated. The limit to the smallest usable crystal size in SMX is the ability to index each data frame, since frames collected from very small crystals will have so few diffracted photons that Bragg peaks will not be obviously identifiable. Because successfully indexing a frame usually requires at least 20–30 resolvable Bragg peaks, under conventional processing schemes data frames that are too weak to identify this number of Bragg peaks would be discarded.

Instead of determining the orientation of each data frame individually, the expand–maximize–compress (EMC) algorithm (Loh & Elser, 2009 ▸) seeks to reconstruct a consistent three-dimensional intensity model using all the data frames simultaneously. The EMC algorithm treats the orientation of each data frame as a probability distribution conditional on the current model and iteratively updates the model by maximizing the associated likelihood function. The validation of its probabilistic modeling of orientations has been demonstrated in many proof-of-concept experiments (Loh *et al.*, 2010 ▸; Philipp *et al.*, 2012 ▸; Ayyer *et al.*, 2014 ▸, 2015 ▸; Ekeberg *et al.*, 2015 ▸; Wierman *et al.*, 2016 ▸), even in some cases where the number of collected photons per frame is extremely low. This success has motivated us to apply the EMC algorithm to SMX to push the limit of usable crystal sizes.

This study is the latest of a series of proof-of-concept table-top experiments with increasing complexity to test the applicability of the EMC algorithm to SMX. We simulated the data frames collected in an SMX experiment by taking a large volume of data frames of very brief exposures from a large hen egg white lysozyme (HEWL) crystal with a dim laboratory X-ray source and a fast-framing mixed-mode pixel array detector (MM-PAD) (Tate *et al.*, 2013 ▸). In contrast to our previous work (Wierman *et al.*, 2016 ▸), where the crystal was rotated about a single axis, the data frames used in this study were collected from a crystal rotated about two orthogonal axes continuously to sample a greater portion of the rotation space. The crystal intensities were reconstructed using the discrete three-dimensional rotation samples lying in this rotation subset, so our method applies to randomly oriented frames by replacing the rotation subset with the whole three-dimensional rotation space. However, like the full exploration of the rotation space, this rotation sampling results in a cubic growth with resolution in the memory and time scaling of the EMC algorithm, which makes the two-axis problem more difficult than its single-axis counterpart. To remedy this problem, we developed computing schemes that greatly reduce the memory usage and computation time. With no input information on the orientation of each data frame, the EMC algorithm successfully reconstructed the Bragg reflections to 2.27 Å resolution. This result further paves the way for EMC-based SMX experiments.

This paper is organized as follows. In §2, we present the details of the experiment, an overview of the EMC algorithm and other aspects of the data processing. In particular, we introduce a local update scheme to speed up the EMC algorithm at high resolution. In §3, we examine the sparsity of the data frames input to the EMC algorithm and the results of the intensity reconstruction. In the appendices, we describe a memory-efficient parallel implementation of the EMC algorithm[App appa] and quantify the speed-up of the local update scheme in practice[App appb].

## 2. Materials and methods   

### 2.1. Setup of rotation axes   

**Figure 1:**
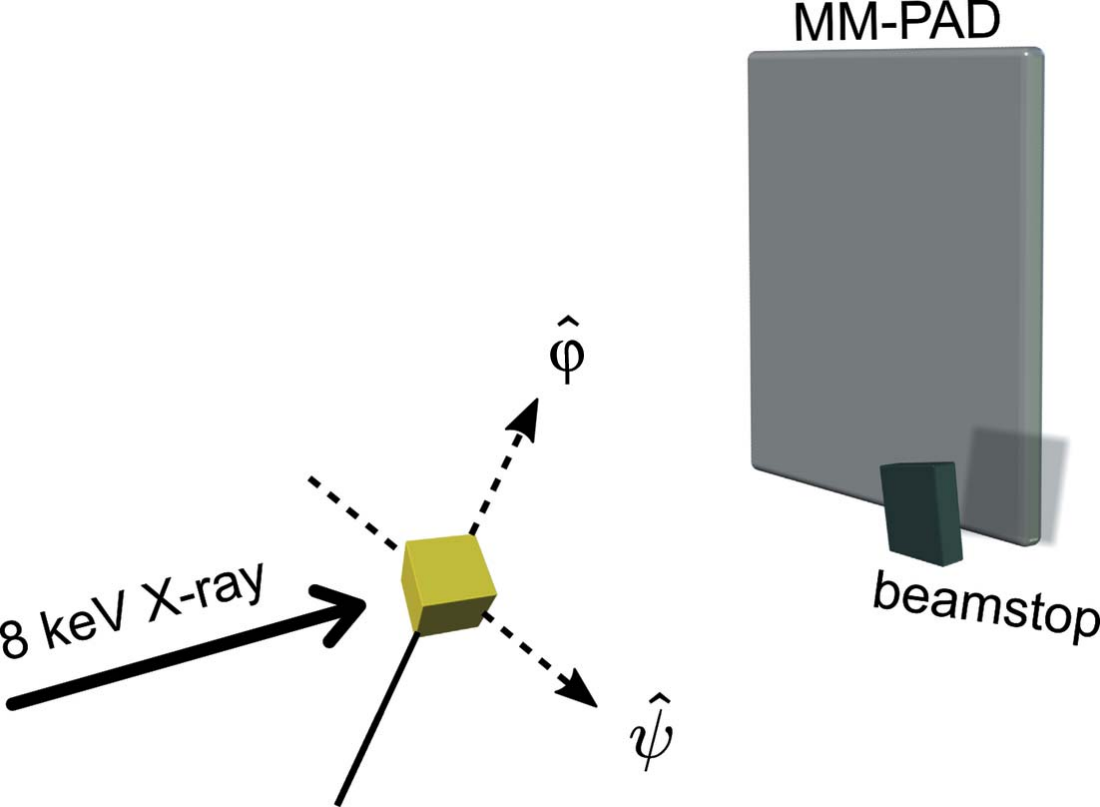
A simplified schematic of the experimental setup with two orthogonal rotation axes. The beam incidence is perpendicular to the ψ axis and the MM-PAD, and the main beam is blocked by the beamstop. The crystal is rotated in increments of 0.1° about the ψ axis, with the data frames recorded by the MM-PAD when φ traverses 360° continuously at each value of ψ. The figure is not drawn to scale.

In order to sample a greater portion of the rotation space than a single rotation axis does (Wierman *et al.*, 2016 ▸), two orthogonal rotation axes were built by fixing the φ rotation stage (Newport URS100) perpendicularly to the ψ rotation stage (Newport UE17CC), as schematically shown in Fig. 1 ▸. In the present experiment rotations about the ψ axis bring the φ rotation stage into alignment with the X-ray beam, so the ψ rotation was limited to 18° to avoid blocking the beam and collision with the detector. This limited angular range of ψ is acceptable because, as described in §2.4.2, the solution to the two-axis problem is readily generalized to full three-dimensional rotations even over this limited angular range. Using a microscope, the rotation axes were adjusted to intersect perpendicularly and their intersection was centered within the X-ray beam using a fluorescent fiber, whose position was recorded for the subsequent sample centering.

### 2.2. Sample preparation   

The protein crystallization technique followed is similar to that described by Wierman *et al.* (2016 ▸). Lyophilized hen egg white lysozyme powder (Sigma, Saint Louis, MO, USA) was dissolved in deionized water to 25 mg ml^−1^ without further purification. Crystals were grown at room temperature (293 K) by the hanging-drop diffusion method by mixing 2 µl of protein solution with 2 µl of reservoir solution containing 1.0 *M* sodium chloride and 0.1 *M* sodium acetate pH 4.5. Crystals appeared after a few days of growth with dimensions 0.4 × 0.4 × 0.6 mm. Using a large-opening pipette fixed on end with a small length of poly(ethylene terephthalate) capillary (outside diameter = 864 µm, wall thickness = 25.4 µm; Advanced Polymers, Salem, NH, USA), a crystal was then retrieved from the crystallization droplets and positioned approximately 22 mm from one end of the capillary. This provided enough remaining length of the capillary on the opposite end to add a small amount of mother liquor solution near the crystal to maintain hydration *via* vapor diffusion during data collection. Mother liquor directly surrounding the crystal was carefully removed from the 22 mm end of the capillary with a paper wick (Hampton Research, Aliso Veijo, CA, USA), and the ends of the capillary were sealed with vacuum grease. The capillary containing the crystal was then mounted on a Hampton Research pin base and attached to a goniometer for data collection.

### 2.3. Data collection   

The X-ray diffraction patterns were collected from the capillary containing a single HEWL protein crystal centered at the intersection of the two orthogonal rotation axes and illuminated by a 0.5 × 0.5 mm Cu 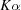 X-ray beam (1.54 Å wavelength). The X-rays were generated from a rotating anode machine set to 36 kV and 50 mA (Rigaku RU-H3R) and focused using Ni-coated Franks mirrors placed 1 m from the sample with a divergence of 1 mrad and a flux of 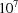
 photons per second. The beam incidence was perpendicular to the ψ axis and the MM-PAD, and the sample-to-detector distance was 60 mm. The center of the beam was placed in one corner of the active area of the MM-PAD, giving a resolution of 2.0 Å in the opposite corner. A pin-diode beamstop was used to prevent the direct beam from striking the MM-PAD during data collection.

The capillary and crystal were rotated about the ψ axis from 0 to 17.9° and then from −18.0 to −0.1° in increments of 0.1°. At each value of ψ, the capillary and crystal were rotated by 360° about the φ axis continuously at a constant angular velocity of 0.5° per second. The MM-PAD collected images at a framing rate of 4 ms per frame in each revolution of φ, which gave an oscillation angle of 0.002° per frame. After the average dark signal had been subtracted and the chip-to-chip global response adjusted, pixel counts were thresholded to avoid false positives. The thresholded pixel counts were then quantized to photon counts by dividing with a known gain and rounding to the nearest integer. Only counts from pixels with at least one photon hit were recorded during data collection to reduce the file size for storage and allow more images to be recorded with the available disk space.

Owing to radiation damage and possible dehydration of the crystal, we only kept the data frames recorded at ψ ranging from 0 to 15.9° to pass on to processing. We also discarded frames that did not record any photons, which was possibly caused by glitches of the rotating anode. To simulate the signal level of an SMX experiment, we further collapsed every 100 successive frames that did not contain any discarded frames, since they were recorded when the crystal was rotated continuously in φ at a fixed value of ψ. We note that an intensity reconstruction was attempted by collapsing every 30 successive frames, but the Bragg reflections could not be reconstructed beyond 3 Å. The collapse of every 100 successive frames gave us 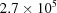 frames with an average of 3000 photons per collapsed frame. These collapsed frames were then passed to the EMC algorithm for intensity reconstruction, though their relative orientations were unknown to the algorithm.

It was discovered after data had been collected and the apparatus disassembled that the crystal was of poor quality. The actual Bragg spot intensities obtained by summing adjacent frames with their known relative orientations cannot be phased to produce a high-resolution structure even though the Bragg peaks do extend to high resolutions. The goal of the experiment, however, was not to solve the well known lysozyme structure but rather to demonstrate that the EMC approach can reconstruct the intensity map in the two-axis case. Because the quality of the reconstructed intensities can be assessed by comparing with the actual intensities, the goal of the experiment could be met even though the crystal was of poor quality for solving a structure.

### 2.4. Intensity reconstruction   

#### 2.4.1. EMC algorithm   

The unoriented data frames were merged into a three-dimensional intensity map iteratively with the EMC algorithm (Loh & Elser, 2009 ▸). Each iteration of the algorithm consists of three steps: expand (E), maximize (M) and compress (C). Consider a reconstruction problem with 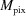 detector pixels, 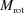 rotation samples, 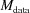 data frames and *N* average photons per frame. Starting with an initial intensity model 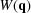, where 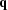 denotes the spatial frequency, the E step calculates the average photon number 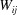, measured at pixel *i* from 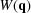 when the crystal has orientation 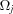. With the data frames represented by 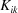, the photon count recorded at pixel *i* in frame *k*, the matrices 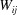 and 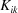 are cross correlated in the M step to evaluate the conditional probability 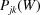 that frame *k* was measured at crystal orientation 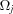 based on the current intensity model *W*. Assuming Poisson statistics, 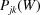 is given by 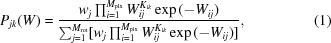
where 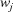 is the fraction of the continuous rotation group assigned to sample 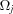. The algorithm subsequently maximizes the expectation value of the log-likelihood function over 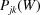 by updating the model according to the rule 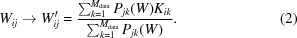
The C step maps 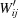 back to the reciprocal space to form a new intensity map 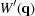 to ensure consistency among all the tomograms 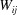 calculated in the next iteration. The algorithm then takes 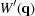 as the initial intensity model 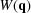 of the next iteration and repeats the iterations until 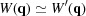.

#### 2.4.2. Reference intensity map, rotation sampling and initial seeding   

Although the relative orientations of the data frames were not passed to the EMC algorithm, we can use them to construct a ‘reference’ intensity map to compare with the reconstructed intensity map. The data frames were mapped to the reciprocal space to form a three-dimensional intensity map according to their relative orientations when recorded. The reciprocal lattice of the crystal is embedded in the intensity map and differs from the laboratory frame by a global rotation 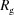. We determined 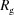 by segmenting out the Bragg peaks (Wierman *et al.*, 2016 ▸) and then applying indexing (Steller *et al.*, 1997 ▸) to the peaks. The intensity map was subsequently rotated by 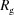 to align with the laboratory frame, and this aligned intensity map is what we call the reference intensity map.

We generated the discrete rotation samples using quaternions (Loh & Elser, 2009 ▸), where the angular resolution 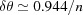 is specified by the order 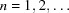. In this study, we confined the rotation samples to those in the subset of rotation space explored by the rotated crystal. The range of the subset in the laboratory frame was found by applying the global rotation 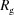 obtained above to the relative orientations between the data frames, though we need to stress that the orientation of each data frame within the subset was unknown to the EMC algorithm. This choice of rotation samples makes the solution to the two-axis problem directly applicable to the randomly oriented frames in real SMX experiments, where the rotation subset is replaced with the whole three-dimensional rotation space.

We seeded the initial intensity map with small three-dimensional Gaussian peaks of random height at each predicted Bragg position, with the lattice constants given by the indexing process mentioned above. In real SMX experiments with a small beam, this information can be obtained from indexing the pseudo-powder patterns. No symmetry was imposed in either the seeding or the reconstruction process.

#### 2.4.3. Local update scheme   

Owing to the exhaustive search in rotations, an EMC reconstruction is usually challenged by its poor time and memory scaling, which are both proportional to 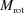. Resolving peaks at high resolution becomes especially difficult, since 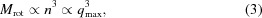
where 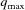 denotes the highest resolvable spatial frequency. Here we propose an update scheme to speed up the EMC algorithm at high resolution, and a parallel implementation that alleviates the memory burden is discussed in Appendix *A* [App appa].

To understand how to speed up the EMC algorithm, we first review how an EMC reconstruction converges in qualitative terms. The peaks at low resolution of the intensity map are reconstructed first owing to the strong diffraction signal at low *q*. These low-resolution peaks hence give each data frame a great preference for certain orientations, and the intensity map is refined about these probable orientations to resolve peaks at higher resolution. With improved signal-to-noise ratio in the intensity map, the convergence gradually proceeds from low *q* to high *q*. This observation shows that the intensity reconstruction has a special feature of locality in orientations: each data frame has high probabilities only at a handful of orientations favored by the low-resolution peaks, while the other orientations with negligible probabilities actually do not contribute to the refinement of the intensity map. Restricting the search to the vicinity of the probable orientations on a per-frame basis can therefore significantly reduce the computation time.

The computing scheme that we call the local update scheme takes advantage of the locality in orientations to speed up the convergence of the intensity reconstruction, and we hereafter refer to the scheme discussed in §2.4.1 as the standard update scheme. The local update scheme consists of two major parts: the calculation of the probable orientation list and the refinement of the intensity map. Starting with a converged low-resolution intensity model 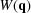 and a coarse rotation sampling 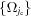 of order 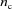, the local update scheme calculates the probabilities 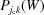 according to equation (1). The probable orientation list is represented by the binary matrix 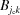 with 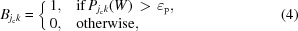
where 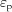 is a pre-defined threshold.

In the second part, the intensity map is refined using a fine rotation sampling 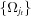 of order 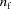 without calculating all the elements of 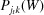. For each coarse rotation sample 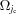, we define its neighborhood as the subset of rotation space that is closer to 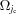 than to any other samples, and assign the fine rotation samples 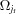 that lie in this subset as the neighbors of 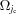. This mapping is stored as a matrix 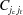, where 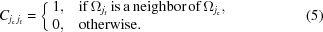
The intensity map is then refined in the same way as in the standard update scheme, with the exception that only the entries of 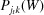 that satisfy the conditions 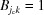 and 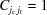 are calculated while the others are set as zero. We hence restrict the calculation of 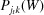 to the neighbors of the probable coarse rotation samples in each data frame. The probable orientation list, or equivalently the binary matrix 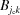, is only recalculated after the intensity map converges to allow a global search over all the coarse rotation samples. The refinement then continues with the updated matrix 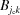. The whole process terminates when the update of the probable orientation list stops changing the intensity map.

Restricting the search in orientations saves a great amount of computation because calculating the probability matrix is the most time-consuming part of the EMC algorithm. A simple estimate (see Appendix *B* [App appb]) shows that the local update scheme can achieve a speed-up by tens to hundreds of times in practice. In addition, the matrices 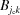 and 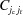 are both sparse, so they barely add any burden to the memory usage. Since the local update scheme places no special focus on the Bragg peaks, it is also applicable to single-particle imaging.

The idea of our local update scheme is similar to the sparse update scheme proposed by Neal & Hinton (1998 ▸), which speeds up the expectation maximization algorithm by freezing the probabilities of improbable values in most of the iterations and only updating them once every many iterations. The only difference is the specific property of locality in our intensity reconstruction application, which allows us to search in a finer grid about the probable coarse rotation samples to refine the intensity map at high resolution. Nonetheless, we need to stress that the only reason to adopt the local update scheme is to speed up the reconstruction at high resolution. The likelihood function maximized in each local update iteration cannot exceed its counterpart when the whole rotation group is explored.

### 2.5. Integration   

After a converged intensity map had been obtained, the reflections were summed over ellipsoidal windows centered at each Bragg position and aligned with the reciprocal lattice. We used the average of the neighboring voxels outside each ellipsoidal window to estimate the background level of each reflection. Reflections with their ellipsoidal windows intersecting the detector gaps or the boundary of the intensity map were considered as partial peaks and rejected.

## 3. Results   

### 3.1. Sparsity of data frames   

**Figure 2:**
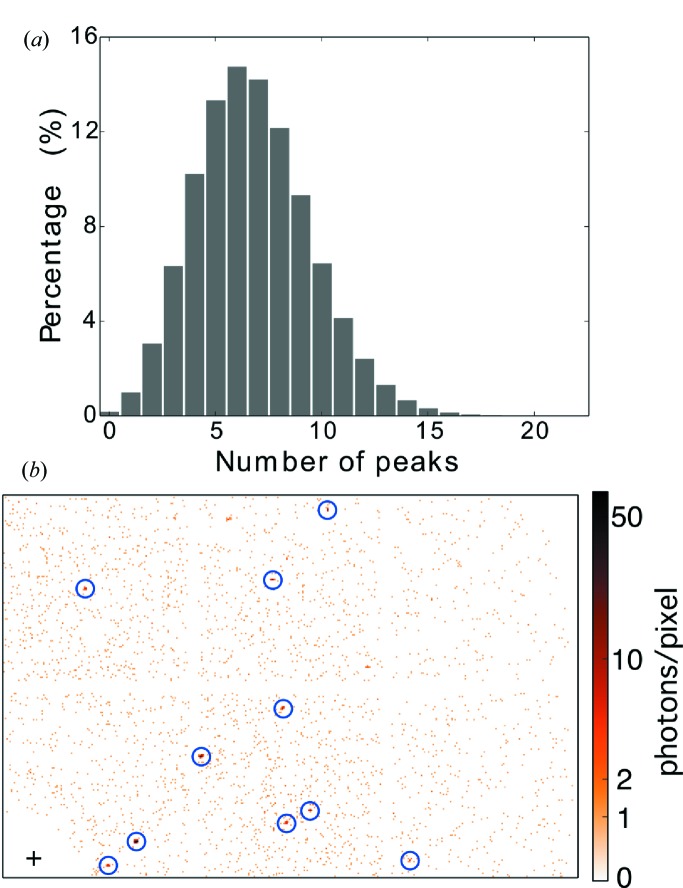
(*a*) Histogram of the number of peaks per collapsed frame, which is the sum of 100 successive frames in the raw data. A patch with more than two connected pixels and an average of no less than two photons per pixel is identified as a peak. (*b*) A random selection of the collapsed frames, with identified peaks marked with blue circles. The cross denotes the beam center, and the resolution at the upper right corner is about 2 Å.

To show the sparsity of the collapsed data frames described in §2.3, we counted the number of peaks per frame with the criterion that a peak has more than two connected pixels and an average of no less than two photons per pixel. As shown in Fig. 2 ▸, most of the frames do not have enough peaks to meet the requirements of conventional indexing methods (at least 20–30), even with this generous criterion for peak finding.

Following the calculation by Holton (2009 ▸), we also estimated the energy absorbed by the crystal over the exposure of one collapsed frame, assuming that protein crystals have the same mass energy absorption cross section as water. Our calculation showed that an 8 µm^3^ crystal would have endured a 0.2 MGy radiation dose if it had scattered the same number of photons as our large HEWL crystal during this period. This dose is within the lifetime of protein crystals at room temperature if the radiation is delivered quickly (Owen *et al.*, 2012 ▸), so the signal level in our study should be comparable to that in a real SMX experiment.

### 3.2. Intensity reconstruction   

Given the 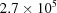 collapsed frames, we started an EMC reconstruction from the randomly seeded model described in §2.4.2 using the standard update scheme and a rotation sampling of order 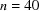. Only data up to 3 Å were used at this stage because our goal was to quickly obtain a converged intensity map at low resolution. After the intensity map had converged, we took its probability distribution and assembled all the data frames to form an intensity map using equation (2) to include data up to 2 Å resolution. This intensity map was then used as the initial model of the local update scheme using rotation samplings of orders 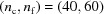 for refinement. Different pairs of orders 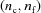 with increasing angular resolutions were sequentially used in the local update scheme to extend the peak convergence to high resolution.

**Figure 3:**
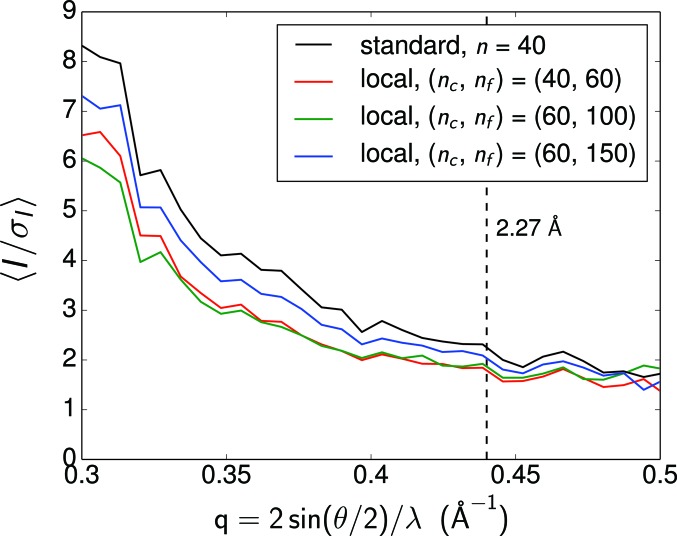
The average signal-to-noise ratio of the integrated reflections from the converged intensity maps at different stages of the reconstruction. The increase of 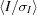 at high *q* indicates the reconstruction of high-resolution peaks. The 2.27 Å resolution determined by CC* is marked by the black dashed line.

Fig. 3 ▸ shows the average signal-to-noise ratio 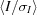 of the integrated reflections from the converged intensity maps at different stages of the reconstruction. We first see that 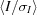 dropped at low resolution while it remained at similar levels at high resolution when moving from the standard update scheme of 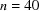 to the local update scheme of 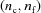 = (40, 60). The lack of improvement at high resolution indicates that the current angular resolution of the local update scheme still cannot resolve high-resolution peaks. On the other hand, the inclusion of data beyond 3 Å slightly disrupted the original probability distribution, which in turn reduced 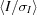 at low resolution. The improvement of 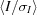 when increasing the angular resolutions signals the reconstruction of high-resolution peaks and justifies the local update scheme.

**Figure 4:**
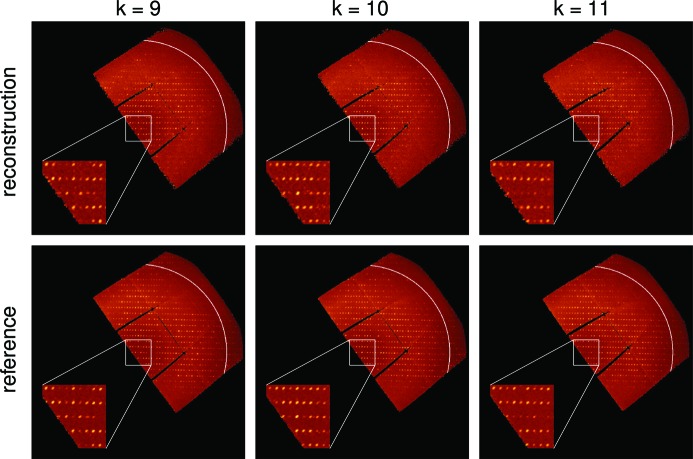
Slices of the reconstructed and reference intensity maps in the *hl* plane at constant values of *k* of the reciprocal lattice. Even without imposing any symmetry in the process of seeding or reconstruction, the converged intensity map still follows the reflection condition 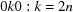 required by the space-group symmetry 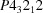 of the HEWL crystal (see insets). The 2.27 Å resolution determined by 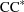 is marked by the arcs in white. The mapping into reciprocal space transforms the detector gaps (Tate *et al.*, 2013 ▸) into curves.

With the converged intensity map from the local update scheme of 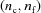 = (60, 150) as our final intensity reconstruction, Fig. 4 ▸ compares the slices of the reconstructed and reference intensity maps perpendicular to the *k* axis of the reciprocal lattice. Although we did not impose any symmetry in the process of seeding or reconstruction, the converged intensity map still follows the reflection condition 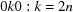 required by the space-group symmetry 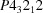 of the HEWL crystal (Hahn, 2006 ▸), which demonstrates the success of the EMC reconstruction. We note that the discrepancy between the two intensity maps in high-resolution peaks is consistent with the low signal-to-noise ratio at high resolutions (see Fig. 3 ▸). Because the photons contributing to the high-resolution shells were mostly collected by the upper left corner of the MM-PAD (Fig. 1 ▸), the resulting lower signal-to-noise ratio made the orientation reconstruction more challenging in this region.

**Figure 5:**
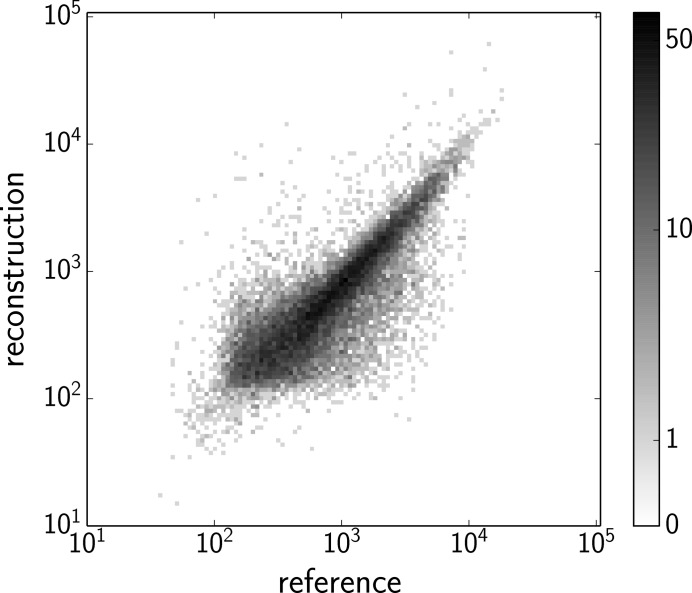
Scatter plot comparing the integrated reflections from the reconstructed and reference intensity maps. Reflections with the signal-to-noise ratio 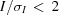 are excluded from the plot. The linear correlation shows the agreement between the two intensity maps.

A further comparison is shown in the scatter plot of the integrated reflections from the reconstructed and reference intensity maps (Fig. 5 ▸), which excludes the reflections with the signal-to-noise ratio 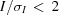. The linear correlation of the reflections shows the consistency of the two intensity maps. Using 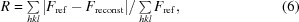
where 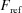 and 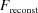 are the structure factors calculated from the reference and reconstructed intensity maps, respectively, we quantify the discrepancy between the two sets of integrated reflections as 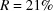, where the reflections with 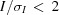 are also excluded. This larger discrepancy than 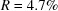 obtained by Wierman *et al.* (2016 ▸) could be caused by the adverse influence of the background scatter and the exploration of a much larger rotation subset than a single rotation axis. By summing the total photon counts of both the integrated and the partial peaks, we estimated the fraction of photons coming from the background and diffuse scatter as ∼90%. We expect to improve the quality of our reconstruction and push the limit to sparser data frames by reducing the background scatter and using a larger detector to gain more information to assist orientation reconstruction.

**Figure 6:**
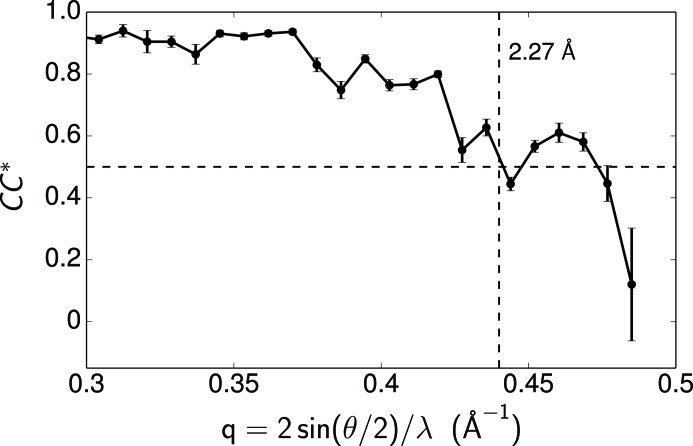
The distribution of 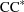 as a function of spatial frequencies. The resolution of the reflections is determined as 2.27 Å by a threshold 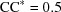. The error bars are estimated by repeating the random separation of reflections 1000 times, while the ups and downs in 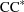 result from the binning in resolution shells.

Another way to assess the quality of the reconstruction is through the calculation of 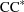, the correlation coefficient of the observed reflections with the underlying true signal (Karplus & Diederichs, 2012 ▸). We first randomly separated the symmetry-related reflections of each unique reflection into two subsets and then calculated the correlation coefficient 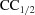 between the average intensities of the two subsets in different resolution shells. Under the assumption that the errors of the two subsets are independent, identically distributed and free from the errors of the true signal, the value of 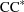 can be estimated from 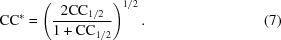
The distribution of 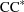 as a function of spatial frequencies is shown in Fig. 6 ▸, with the error bars estimated by repeating the random separation of reflections 1000 times. The large error bar in the highest-resolution shell shows the low correlation between the intensities of the two subsets, which is consistent with the low signal-to-noise ratio at high resolution. We determine the resolution of the reconstructed reflections as 2.27 Å by a threshold 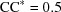. This choice is consistent with the resolution where the average signal-to-noise ratio of our final reconstructed intensity (the black curve in Fig. 3 ▸) drops to 2. We note that the value of the correlation coefficient is dominated by the stronger peaks in each resolution shell. Therefore, CC* can still have moderate values at high resolutions even if some low-signal peaks are not resolvable, as indicated by the discrepancy between the two intensity maps in high-resolution peaks in Fig. 4 ▸.

## 4. Conclusion   

The results of this study show that the limit to the usable crystal sizes in current SMX experiments could be relaxed by employing the EMC algorithm. Because the algorithm leverages the data redundancy arising from the common arcs between pairs of diffraction patterns, the intensity reconstruction is feasible even though each frame may not contain sufficient information to be oriented individually. The computing schemes we have developed in this article further alleviate the computational requirements of the EMC reconstruction, which makes EMC-based SMX experiments more practical.

The fact that the EMC algorithm was able to reconstruct the actual X-ray intensity incident on the detector, irrespective of crystal quality, illustrates the generality of the algorithm. The algorithm has no ‘knowledge’ of what is being reconstructed. Everything in the detected X-ray field – background, diffraction spots, diffuse scatter *etc.* – is reconstructed.

Several issues remain to be addressed to put EMC-based SMX experiments into practice. In contrast to the randomly sampled crystal orientations in real SMX experiments, the data frames in this study were taken from a crystal rotated continuously. From our past experience, this difference in orientation sampling should not affect the EMC reconstruction as long as the random orientation sampling size is large enough. We estimate that 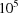
–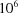 data frames are required, which amounts to a data collection time of within a day when a 10% single-crystal hit rate and an exposure time of 10 ms per frame are assumed.

In this paper we used a large single crystal in various orientations to emulate the data expected from multiple small crystals. The obvious next step towards practical application of the method is to try the EMC algorithm on data from multiple small crystals. It will be necessary to experimentally determine the severity of difficulties arising from sources including varying crystal diffraction quality and occasional multiple crystals in the beam. We expect to incorporate metrics such as the normalized surprise function (Munke *et al.*, 2016 ▸) into the EMC algorithm to estimate the reliability of each frame based on the current intensity model and reject frames containing multiple lattices. To tackle the frame-to-frame crystal size variation, the EMC algorithm also needs to calculate the relative contribution of each frame to the intensity model iteratively. As indicated by Loh *et al.* (2010 ▸), the intensity model could be updated by maximizing the likelihood function with respect to the relative crystal sizes and the crystal orientations alternately, with the cost of doubling the computation time.

The last issue is the background scatter. In principle, the EMC algorithm is able to deal with stable background by modifying the conditional probability calculation. However, background reduction by improving the experiment becomes necessary when the frame-to-frame variation in background is significant. With the above issues considered, the analysis of SMX data would involve first reconstructing a low-resolution intensity map using the standard EMC update scheme, and then refining the high-resolution peaks using the local update scheme because of its computational efficiency.

## Appendix A Memory-efficient parallel implementation

Here we describe a memory-efficient parallel implementation of the EMC algorithm, which is applicable to both the standard and local update schemes. The memory usage of the EMC algorithm is dominated by the matrices  and , with sizes of  and , respectively. Since , the required memory rapidly becomes intractable, for example, hundreds of terabytes (TB), even to resolve peaks at moderate resolutions (Ayyer *et al.*, 2016 ▸).

However, only a small portion of the entries of  are significant according to our discussion in §2.4.3, so we can treat  as a sparse matrix to reduce the required memory. This amounts to the fact that a given data frame only has non-negligible probabilities at a small fraction of the orientations, unless the signal level is as weak as only several photons per frame. In our implementation, we distribute blocks of data frames (ranges in *k* index) to different processors, each of which holds the same copy of the intensity map . The algorithm strides through the  rotations in steps of size  and calculates  and  in each step. Here we define  as

and each processor dynamically updates the value of  when walking through all the orientations. From the inequality

the entries of  are saved only when the ratio  exceeds the pre-defined threshold , and this condition is checked for all the saved entries every time the value of  is updated. After going through all the rotation samples, the algorithm calculates the significant values of  by normalizing the saved entries of  over orientations. Subsequently, we update the tomograms  also in steps of size  over all the orientations, and map  back to the copy of the updated intensity map  held by each processor after each step. Finally,  is reduced among all the processors to complete the iteration.

As a result, the memory scaling of  and  is reduced to  and , respectively, where  denotes the average number of probable orientations per frame and is governed by the threshold  and the signal level. The memory usage can be limited to only tens of gigabytes even when using an extremely fine rotation sampling, since the dominant memory scaling is independent of .

## Appendix B Speed-up of the local update scheme

The most time-intensive part of a standard EMC update is to calculate the probability matrix , which is proportional to . In a local update scheme with  and  rotation samples, this proportionality becomes , where  is the average number of the probable coarse rotation samples per frame and  is the average number of neighbors each coarse rotation sample has. Consider a local update scheme that recalculates the most probable orientation lists  every  iterations. The speed-up is given by

where the numerator is the time proportionality for  iterations of standard updates using the fine rotation sampling, and  in the first term of the denominator denotes the proportionality for the recalculation of .

The number of rotations that samples the rotation space with order *n* is given by . Consider a local update scheme that uses  and rotation samplings of orders  = (60, 150). Assume that a chosen value of  leads to . Equation (7) gives a speed-up of 156 times.
