# Reconstructing three-dimensional protein crystal intensities from sparse unoriented two-axis X-ray diffraction patterns. Corrigendum

**DOI:** 10.1107/S1600576718000171

**Published:** 2018-02-01

**Authors:** Ti-Yen Lan, Jennifer L. Wierman, Mark W. Tate, Hugh T. Philipp, Veit Elser, Sol M. Gruner

**Affiliations:** a Laboratory of Atomic and Solid State Physics, Cornell University, Ithaca, NY 14853, USA; b Cornell High Energy Synchrotron Source (CHESS), Cornell University, Ithaca, NY 14853, USA; c Macromolecular Diffraction Facility at CHESS (MacCHESS), Cornell University, Ithaca, NY 14853, USA; d Kavli Institute for Nanoscale Science, Cornell University, Ithaca, NY 14853, USA

**Keywords:** X-ray serial microcrystallography, sparse data, EMC algorithm, protein microcrystallography, synchrotron radiation sources

## Abstract

A figure in the article by Lan, Wierman, Tate, Philipp, Elser & Gruner [*J. Appl. Cryst.* (2017), **50**, 985–993] is corrected.

## Abstract 2

Corrigendum to *J. Appl. Cryst.* (2017), **50**, 985–993.

The color code of the curves in Fig. 3 on p. 989 of the article by Lan *et al.* (2017 ▸) is incorrect. The correct code (as shown in Fig. 1 ▸) is as follows:

Topmost (black) curve: local (*n*_c_, *n*_f_) = (60, 150)

Second from top (blue) curve: local (*n*_c_, *n*_f_) = (60, 100)

Third from top (red) curve: standard, *n* = 40

Bottom (green) curve: local (*n*_c_, *n*_f_) = (40, 60)

## Figures and Tables

**Figure 1:**
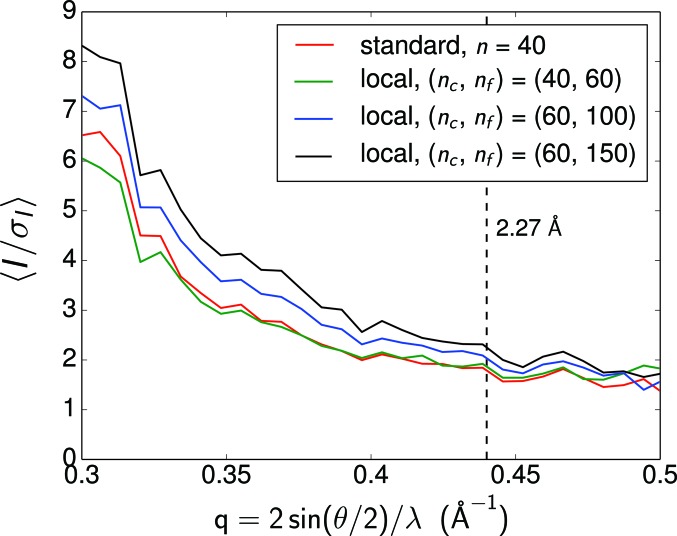
The corrected version of Fig. 3 of Lan *et al.* (2017 ▸). The average signal-to-noise ratio of the integrated reflections from the converged intensity maps at different stages of the reconstruction. The increase of 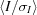 at high *q* indicates the reconstruction of high-resolution peaks. The 2.27 Å resolution determined by CC* is marked by the black dashed line.
